# Chronic restraint stress induces changes in the cerebral Galpha 12/13 and Rho-GTPase signaling network

**DOI:** 10.1007/s43440-021-00294-4

**Published:** 2021-06-11

**Authors:** Katarzyna Rafa-Zabłocka, Agnieszka Zelek-Molik, Beata Tepper, Piotr Chmielarz, Grzegorz Kreiner, Michał Wilczkowski, Irena Nalepa

**Affiliations:** 1 Department of Brain Biochemistry, Maj Institute of Pharmacology, Polish Academy of Sciences, Smętna 12, 31-343 Kraków, Poland; 2 Laboratory of Calcium Binding Proteins, Nencki Institute of Experimental Biology, Polish Academy of Sciences, Pasteura 3, 02-093 Warsaw, Poland

**Keywords:** Chronic stress, Galpha proteins, G-protein-coupled receptors, Mouse brain, Rat brain, Rho GTPases

## Abstract

**Background:**

Evidence indicates that Gα12, Gα13, and its downstream effectors, RhoA and Rac1, regulate neuronal morphology affected by stress. This study was aimed at investigating whether repeated stress influences the expression of proteins related to the Gα12/13 intracellular signaling pathway in selected brain regions sensitive to the effects of stress. Furthermore, the therapeutic impact of β(1)adrenergic receptors (β1AR) blockade was assessed.

**Methods:**

Restraint stress (RS) model in mice (2 h/14 days) was used to assess prolonged stress effects on the mRNA expression of Gα12, Gα13, RhoA, Rac1 in the prefrontal cortex (PFC), hippocampus (HIP) and amygdala (AMY). In a separate study, applying RS model in rats (3–4 h/1 day or 14 days), we evaluated stress effects on the expression of Gα12, Gα11, Gαq, RhoA, RhoB, RhoC, Rac1/2/3 in the HIP. Betaxolol (BET), a selective β1AR antagonist, was introduced (5 mg/kg/*p.o*./8–14 days) in the rat RS model to assess the role of β1AR in stress effects. RT-qPCR and Western Blot were used for mRNA and protein assessments, respectively.

**Results:**

Chronic RS decreased mRNA expression of Gα12 and increased mRNA for Rac1 in the PFC of mice. In the mice AMY, decreased mRNA expression of Gα12, Gα13 and RhoA was observed. Fourteen days of RS exposure increased RhoA protein level in the rats’ HIP in the manner dependent on β1AR activity.

**Conclusions:**

Together, these results suggest that repeated RS affects the expression of genes and proteins known to be engaged in neural plasticity, providing potential targets for further studies aimed at unraveling the molecular mechanisms of stress-related neuropsychiatric diseases.

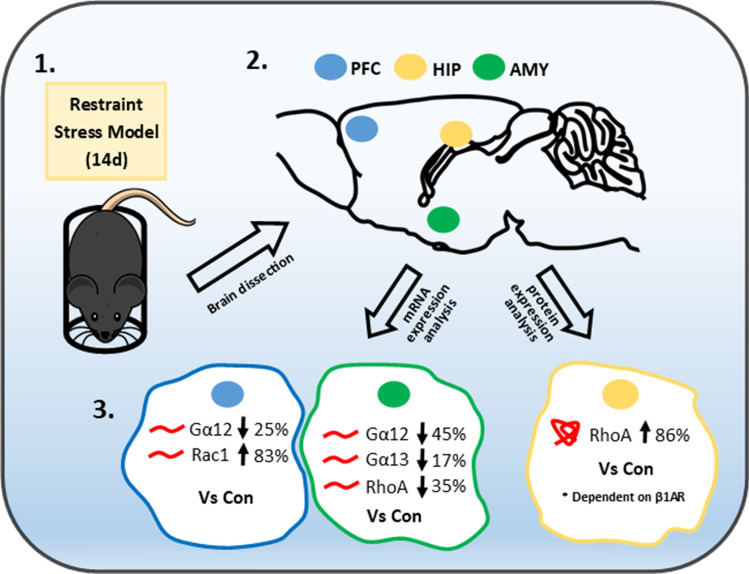

**Supplementary Information:**

The online version contains supplementary material available at 10.1007/s43440-021-00294-4.

## Introduction

Excessive stress is considered an important etiological factor that can disturb the physiological integrity of the organism and contribute to various neuropsychiatric as well as neurodegenerative diseases [1, 2]. The effects of excessive stress can be observed at various signal transduction levels, including changes in the synaptic level of neurotransmitters, their receptors, and intracellular proteins engaged in regulating neuronal activity and structure. Indeed, a growing body of evidence from various stress models indicates that repeated stress affects the brain’s structural and functional plasticity [3]. Changes in plasticity are manifested by brain region-specific alterations in dendritic and synaptic structure [4–7].

Various central nervous system neurotransmitters involved in stress response modulation act through the metabotropic G-protein-coupled receptors (GPCR). The heterotrimeric (αβγ) G proteins perform the role of molecular switches between these receptors and intracellular signaling pathways. Of the four major families of G proteins (classified after their α subunits as Gαs, Gαo/i, Gαq/11 and Gα12/13), the regulation of the Gα12/13 proteins activity in the brain is the least understood. They can act as preferential effectors of the GPCRs coupling, typically to other Gα subunits, like in the case of α1-adrenergic receptors, muscarinic M_1_ or M_3_ receptor, or 5-HT_2C_ serotonergic receptor [8] or provide a sole pathway for downstream signaling, like in the case of 5-HT_4_ or 5-HT_7_ serotonergic receptors [9]. Moreover, in vivo treatment with opioid receptor agonists or 5-HT_7_ receptors antagonists may affect Gα12/13 mRNA expression [10–12].

Gα12/13 proteins have been demonstrated to regulate various cellular processes via cooperation with Rho-family GTPases (e.g. RhoA, Rac1, or Cdc42), known as regulators of actin cytoskeleton remodeling [13, 14]. In neurons, Rho-GTPases modulate dendritic reorganization, including dynamic alterations of dendritic spines, which are crucial elements of synaptic plasticity [15, 16]. In particular, RhoA and Rac1, the best known Rho-GTPases, have been shown to influence neuronal morphology. RhoA contributes to dendritic arbor simplification, whereas Rac1 is primarily involved in the maintenance of dendritic spines and dendritic arborization [17].

Structural changes in neurons are among the most pronounced effects of stress and are probably a key sign of stress-related pathology [18, 19]. However, the molecular mechanisms involved in stress-induced brain neuroplastic changes are still poorly understood. Given the vital role of the proteins mentioned above in the regulation of neuronal morphology, this study aimed to investigate whether repetitive restraint stress (RS), known to induce morphological alterations [5, 20], influences Galpha 12/13 and Rho-GTPase cross-linked signaling proteins at the level of mRNA in the prefrontal cortex (PFC), hippocampus (HIP) and amygdala (AMY) in mice, and on the protein level in the rat HIP. Furthermore, the involvement of β1 adrenergic receptors (β1AR) in the stress pathology and the therapeutic potential of β1AR blockade were analyzed.

## Materials and methods

### Animals

The experiments were performed on adult C57BL/6N male mice (12-week-old) and adult Wistar Han male rats weighting 343–425 g (1 day RS experiment) and 285–340 g (chronic RS). Animals (Charles River, Germany) were housed in standard conditions at room temperature 21 ± 1 °C, 40–50% humidity, and 12 h light/dark cycle. Food and water were available ad libitum, except during immobilization applied to stressed groups.

All procedures were approved by the Local Ethical Commission for Animal Experiments at Maj Institute of Pharmacology, Polish Academy of Sciences in Krakow (Permit number for the experiment involving mice: 789, 30.09.2010, and Permit number for the study on rats: 1106, 22.07.2014) and fulfilled the requirements of the EU Directive 2010/63/EU on the protection of animals used for scientific purposes.

### Stress procedure

Animals were randomly assigned to experimental groups. Stress procedure was performed in light phase of light/dark cycle. The mice from the stress group were restrained in well-ventilated polypropylene conical tubes (50 ml) for 2 h daily for 14 consecutive days. In rats, the 1-day stress paradigm consisted of 3 h daily of RS, while in the case of chronic stress exposure, RS was applied for 4 h daily in the first 2 days and 3 h in the next 12 days. The rats were placed in perforated plastic tubes (6.5 cm inner diameter) of adjustable length. The restraint allowed breathing and limited movements of the head and limbs. After the stress procedure, animals were removed from the restrainers and returned to their home cages. Control (sham) animals were held in their home cages during the stress sessions.

### Betaxolol treatment protocol

To assess the role of β1AR in the studied mechanism and to analyze the therapeutic effect of its inhibition, rats were treated with betaxolol (Alcon, Fort Worth, TX, USA). In the experiment with the 1-day RS paradigm, four groups were generated: sham/SAL (*n* = 7); RS/SAL (*n* = 7); sham/BET (*n* = 6); RS/BET (*n* = 6). In these groups, ‘RS’ and ‘sham’ procedures were applied once, as described in the previous section. Animals received single oral administration (*po*, per os) of betaxolol (5 mg/kg/*po*) (‘BET’ groups) or 0.9% NaCl (0.5 ml/rat) (‘SAL’ groups).

In the experiment with chronic RS, separate four animal groups were generated: sham/SAL (*n* = 7); RS/SAL (*n* = 7); sham/BET (*n* = 5); RS/BET (*n* = 5). In these groups, ‘RS’ and ‘sham’ procedures were applied repeatedly for 14 days, as described earlier. In the 2nd week of RS procedure, immediately after stress, the treatment with betaxolol (5 mg/kg/*po*) or 0.9% NaCl (0.5 ml/rat/*po*) was introduced for ‘BET’ and ‘SAL’ groups, respectively. Rats were treated for 7 consecutive days, from 8 to 14th experimental day. Dose and route of treatment were chosen based on the literature [21] and own preliminary results showing that the treatment protocol had no sedative effect on rats (measured by locomotor activity) and reversed the impact of chronic RS on the rats’ body weight.

### Tissues

All animals were decapitated 24 h after the last stress session. For the purpose of mRNA study the HIP, PFC and AMY were isolated from excised mouse brains, rapidly immersed in 500 μl of RNAlater^®^ (Ambion, USA) and stored first at 4 °C for 24 h and then at − 20 °C until further processing. To assess the protein expression, the HIP was isolated on an ice-cold glass plate, immediately frozen on dry ice and stored at − 70 °C until assayed.

### RNA isolation and RT-qPCR

Total RNA was isolated from the brain tissue using the RNAeasy Mini Kit (Qiagen, Germany) according to the manufacturer’s protocol. The quantity of RNA was determined spectrophotometrically using a NanoPhotometer^®^ (IMPLEN, Germany) at 260 nm. The quality of RNA was assessed based on the 260 nm/280 nm absorbance ratio. Samples of total RNA (1 µg/reaction) were reverse transcribed (RT) to cDNA using the High-Capacity cDNA Reverse Transcription Kit (Applied Biosystems, USA) in a final volume of 20 μl for 2 h at 37 °C. mRNA expression levels of Gα12, Gα13, Rac1 and RhoA were assessed using quantitative PCR (qPCR) with TaqMan^®^ probes (*Gna12 Mm00494665_m1, Gna13 Mm00494667_m1, Rac1 Mm01201657_g1, RhoA Mm01601614_g1*; Applied Biosystems, USA). The PCR reaction mix consisted of 50 ng of cDNA, 1 μl of a specific probe, 10 μl of ready-to-use FastStart Universal Probe Master (ROX) mix (Roche, Germany), and water to a final volume of 20 μl. The qPCR reaction was run in a Chromo4 Real-Time PCR Instrument (MJ Research, USA) with the following thermocycler protocol: 95 °C/10 min activation step, followed by 40 cycles of 95 °C/15 s denaturation and 60 °C/1 min for amplification and quantification. The qPCR reaction for each sample was prepared in two technical replicates. The cycle threshold value (Ct) for each gene of interest was set in the PCR’s exponential phase. Samples were analyzed for relative gene expression using the ΔΔCt method and normalized to beta-2 microglobulin (*B2m, Mm00437762_m1*) as an internal control.

### Protein isolation and immunoblotting

Total protein was extracted through high-speed shaking in plastic tubes with stainless-steel beads in a tissue lyser with 100 μl ice-cold RIPA lysis buffer (Sigma, USA) containing a complete mini protease inhibitor (Roche Diagnostics, USA). After incubation for 30 min, the homogenates were centrifuged at 10,000×*g* for 20 min at 4 °C. The resulting supernatants were collected and subjected to protein analysis using the Bicinchoninic Acid Assay Kit (Sigma, USA). Next, the standard Western blot procedure was conducted, as previously described [22]. Briefly, equal amounts of protein extracts (6–20 μg) were boiled in Laemmli buffer containing 5% β-mercaptoethanol for 5 min, separated through SDS-PAGE (4–15%) and transferred to nitrocellulose membranes. The membranes were blocked with 5% nonfat dry milk in Tris-buffered saline containing Tween-20 (TBST; pH = 7.6) for 1 h at room temperature and incubated with one type of primary antibody against: Gα12, Gα11, Gαq, (1:1000; Santa Cruz Biotechnology, Dallas, TX, USA), RhoA, RhoB, RhoC, Rac1/2/3 (1:1000; Cell Signaling Technology, Danvers, MA, USA), overnight at 4 °C. After three washes with the blocking solution, the membranes were incubated with the appropriate secondary antibodies for 1 h at room temperature, followed by three washes with TBST. Antibody binding was detected using an enhanced chemiluminescence kit (ECL Plus, Pierce, USA). Equal loading of protein per sample was further confirmed after probing with anti-calnexin (CNX) antiserum (1:5000; ADI-SPA-865-F, Enzo Life Sciences, USA). All Western blot analyses were performed at least twice to confirm the results. The chemiluminescence signal was visualized using a Luminescent Image Analyzer Fuji-Las 4000 (Fuji, Japan). Immunoreactive bands were quantified using an image analyzer (ScienceLab, MultiGauge V3.0).

### Data analysis

All presented values were averaged from 5 to 8 animals ± the standard error of the mean (SEM). The mRNA data were analyzed by a one-way analysis of variance (ANOVA) followed by Fisher’s Least Significant Difference (LSD) test, while the protein data by a two-way ANOVA followed by unequal N HSD test using the Statistica 10.0 software (Round Rock, TX, USA). Analyzed results were considered statistically significant when *p* < 0.05.

## Results

### The effects of repeated restraint stress on the mRNA expression of Gα12, Gα13, RhoA, Rac in the mouse PFC, AMY and HIP

**Fig. 1:**
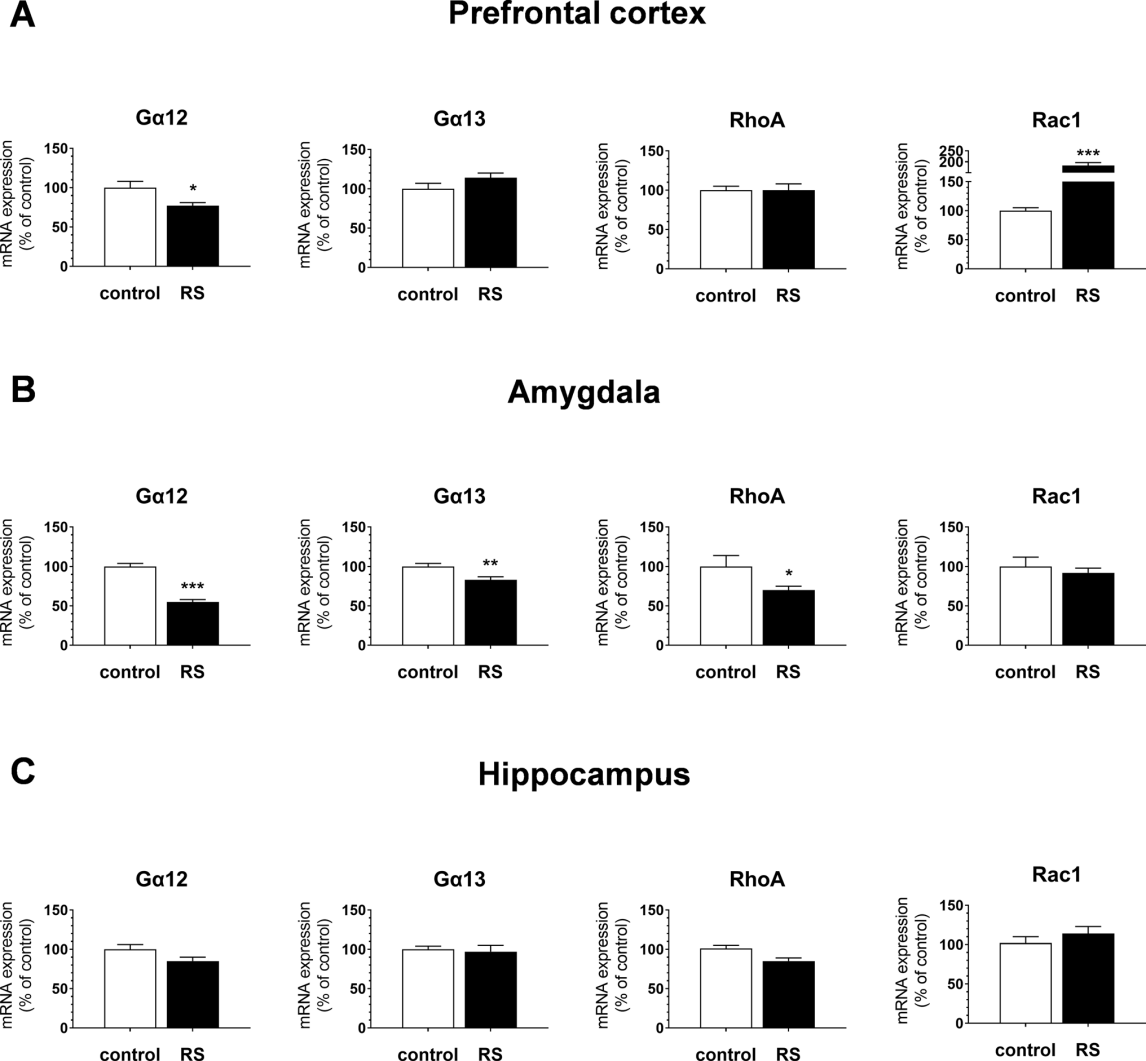
The effects of chronic restraint stress (RS) on the mRNA expression of Gα12/13 and RhoA/Rac1 proteins in the mouse prefrontal cortex (**A**), amygdala (**B**) and hippocampus (**C**). The results of a one-way ANOVA for the RS effects on Gα12 [*F*_1,13_ = 8.33, *p* = 0.012], Rac1 [*F*_1,12_ = 27.94, *p* < 0.001] in the PFC, and on Gα12 [*F*_1,13_ = 76.79, *p* < 0.0001], Gα13 [*F*_1,13_ = 10.37, *p* = 0.006], RhoA [*F*_1,12_ = 5.82, *p* = 0.03] in the AMY. The bars represent the mean ± SEM. Group sizes: PFC: *n* = 6 (control) and *n* = 8 (RS); AMY: *n* = 7 (control) and *n* = 7 (RS); HIP: *n* = 7 (control) and *n* = 8 (RS). *p < 0.05, **p < 0.01, ***p < 0.001 vs control group (Fischer’s LSD test)

Chronic restraint stress-evoked changes in mRNA expression of the analyzed genes that were differently pronounced among the brain regions considered (Fig. 1). In the PFC, compared to the control group, mRNA levels of Gα12 were decreased by 25% [*F*_1,13_ = 8.33, *p* < 0.05], while Rac1 increased by 83% [*F*_1,12_ = 27.94, *p* < 0.001]. The mRNA levels of Gα13 and RhoA remained unchanged in the PFC (Fig. 1A). In the AMY, Gα12 and Gα13 mRNA levels were reduced by 45% [*F*_1,13_ = 76.79, *p* < 0.001] and 17% [*F*_1,13_ = 10.37, *p* < 0.01], respectively. Moreover, in this brain structure, chronic RS reduced the RhoA mRNA levels by 35%, compared to the control [*F*_1,12_ = 5.82, *p* < 0.05], while Rac1 mRNA levels were not affected by chronic RS in the AMY (Fig. 1B). No changes in mRNA expression were found in the HIP (Fig. 1C).

### The effect of stress and its modulation by betaxolol on the GTPase protein expression in the rat HIP

#### RhoA

**Fig. 2:**
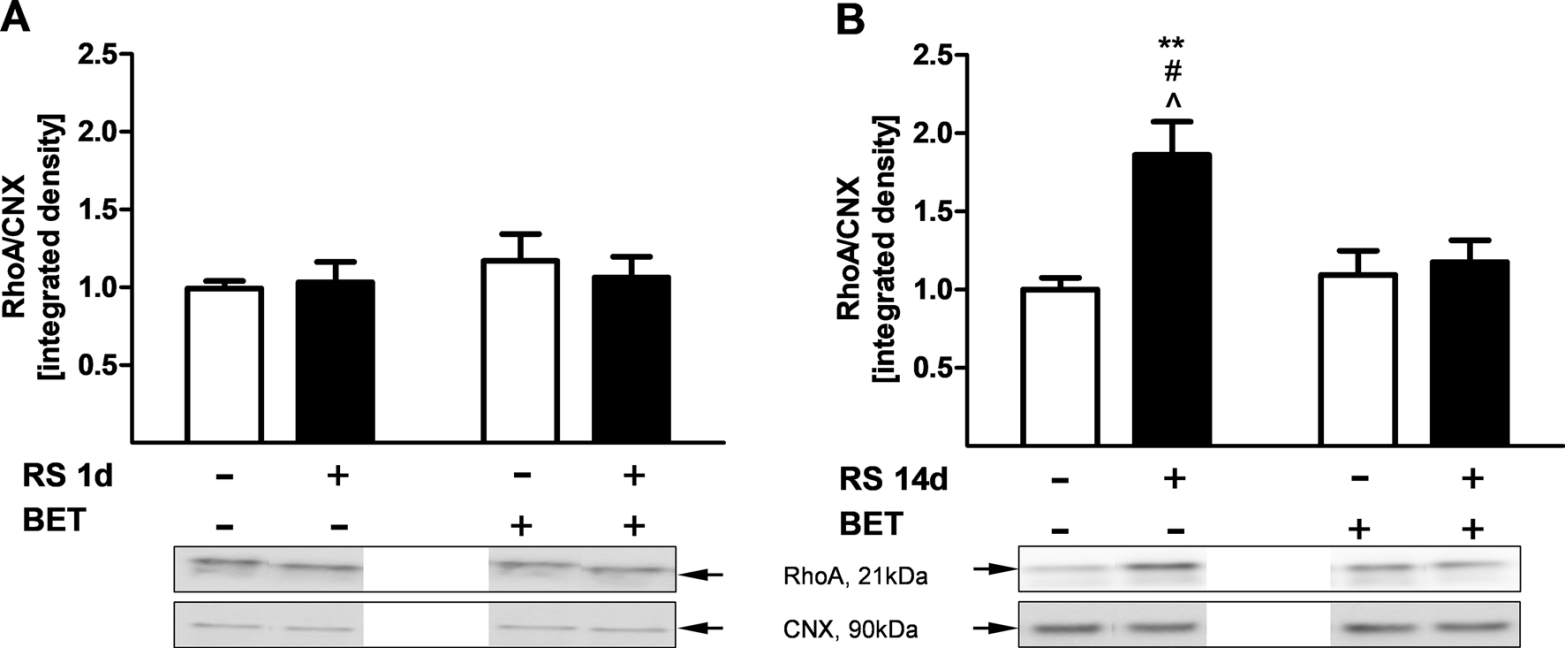
The effects of single (**A**) and chronic (**B**) restraint stress (RS) and betaxolol (BET) (5 mg/kg/*po*) post-stress treatment on the protein expression of RhoA in the rat hippocampus. The bars represent the mean ± SEM. In **A**: single RS effect [*F*(1,20) = 0.07; *p* = 0.80]; single BET effect [*F*(1,20) = 0.65; *p* = 0.43]; single RS × single BET interaction [*F*(1,20) = 0.03; *p* = 0.58]; *n* = 6/each group. In **B**: RS effect [*F*(1,18) = 9.14; *p* < 0.01]; BET effect [*F*(1,18) = 3.57; *p* = 0.07]; RS × BET interaction [*F*(1,18) = 6.25; *p* < 0.05]; group sizes: *n* = 6 (sham/SAL); *n* = 5 (sham/BET); *n* = 6 (RS/SAL) *n* = 5 (RS/BET). **p < 0.01 vs sham/SAL group; ^#^*p* < 0.05 vs sham/BET; ^*p* < 0.05 vs RS/BET group (unequal N HSD test). *CNX* calnexin

A two-way ANOVA revealed lack of influence of single RS [*F*_1,20_ = 0.07; *p* = 0.80] and interaction of single RS × single BET [*F*_1,20_ = 0.03; *p* = 0.58] on the RhoA protein expression in the rat HIP (Fig. 2A). However, chronic RS increased RhoA expression by 86% vs sham/SAL group. The level of RhoA protein increased by chronic RS exposure was normalized when rats received BET in the 2nd week of RS procedure immediately after RS procedure (Fig. 2B). A two-way ANOVA (considering chronic RS–RS 14 days, sham and pharmacological treatment—SAL, BET—as independent factors) used to analyze RhoA levels showed significant effects of RS 14 days [*F*_1,18_ = 9.14; *p* < 0.01] and RS 14 days × BET interaction: [*F*_1,18_ = 6.25; *p* < 0.05]. An unequal *N* HSD test revealed a higher level of RhoA in the chronic RS/sham group in comparison to other groups: sham/SAL (*p* < 0.01), sham/BET (*p* < 0.05) and chronic RS/BET (*p* < 0.05). Betaxolol did not change RhoA expression in the rat HIP after single [*F*_1,20_ = 0.65; *p* = 0.43] and repeated treatment [*F*_1,18_ = 3.57; *p* = 0.07] (Fig. 2).

##### Gα12, Gα11, Gαq, RhoB, RhoC, Rac1/2/3

**Fig. 3:**
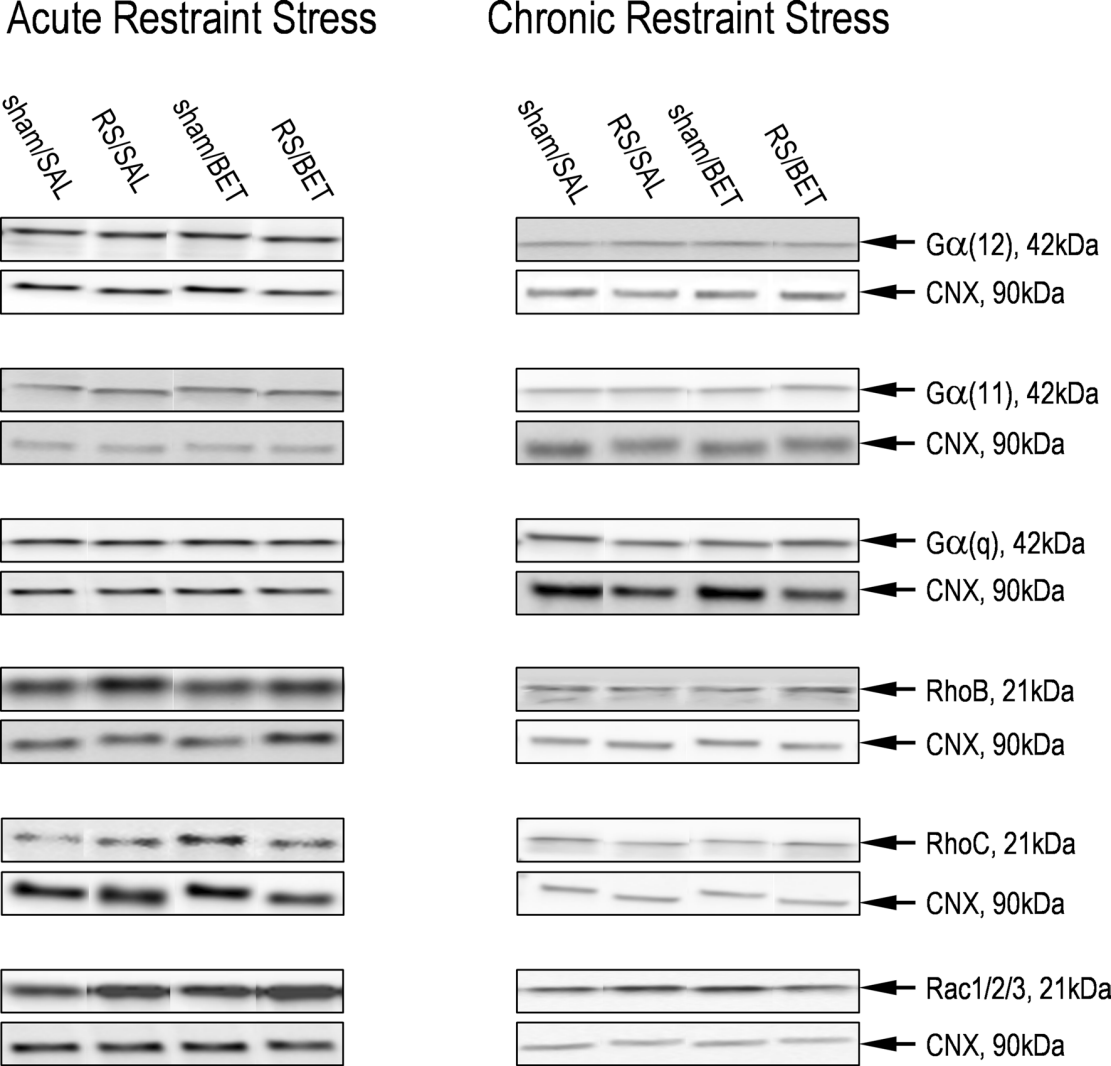
Representative immunoblots that illustrate protein expression of Gα(12), Gα(11), Gα(q), RhoB, RhoC, and Rac1/2/3 in the rat HIP. Left columns—effects of single RS and BET treatment. Right columns—effects of chronic RS and BET treatment. For other descriptions, see Fig. 2

No differences among groups were observed in the protein levels of Gα12, Gα11, Gαq, RhoB, RhoC, Rac1/2/3 in the rat HIP after single and chronic exposure to RS and BET treatment (Table 1; Suppl. Tab. 1; Fig. 3).

**Table 1:** Lack of single and repeated restraint stress effects and betaxolol treatment on the protein expression of Gα(12), Gα(11), Gα(q), Rho B, Rho C, Rac 1/2/3 in the rats’ hippocampus

Name of protein	Acute restraint stress				Chronic restraint stress			
	sham/SAL	RS/SAL	sham/BET	RS/BET	sham/SAL	RS/SAL	sham/BET	RS/BET
Gα(12)	1.00 ± 0.11 *n* = 6	0.79 ± 0.18 *n* = 7	0.98 ± 0.10 *n* = 5	0.96 ± 0.20 *n* = 5	0.97 ± 0.07 *n* = 6	1.13 ± 0.07 *n* = 6	1.04 ± 0.05 *n* = 6	1.08 ± 0.05 *n* = 6
Gα(11)	1.04 ± 0.11 *n* = 6	1.01 ± 0.11 *n* = 7	1.05 ± 0.17 *n* = 5	1.10 ± 0.14 *n* = 5	1.02 ± 0.07 *n* = 5	0.94 ± 0.21 *n* = 5	1.07 ± 0.14 *n* = 6	1.22 ± 0.18 *n* = 5
Gα(q)	1.01 ± 0.07 *n* = 7	1.39 ± 0.26 *n* = 7	1.07 ± 0.10 *n* = 5	1.20 ± 0.11 *n* = 5	0.99 ± 0.08 *n* = 6	0.79 ± 0.06 *n* = 6	0.92 ± 0.10 *n* = 6	0.98 ± 0.08 *n* = 6
Rho B	1.01 ± 0.12 *n* = 6	1.23 ± 0.26 *n* = 6	0.94 ± 0.13 *n* = 6	1.06 ± 0.15 *n* = 6	1.06 ± 0.22 *n* = 7	1.31 ± 0.24 *n* = 7	0.98 ± 0.24 *n* = 5	0.84 ± 0.23 *n* = 5
Rho C	0.99 ± 0.15 *n* = 6	1.06 ± 0.11 *n* = 6	1.11 ± 0.09 *n* = 6	0.91 ± 0.09 *n* = 6	1.01 ± 0.16 *n* = 6	1.08 ± 0.11 *n* = 6	0.77 ± 0.08 *n* = 5	0.96 ± 0.16 *n* = 5
Rac1/2/3	1.03 ± 0.10 *n* = 6	1.15 ± 0.11 *n* = 6	1.11 ± 0.12 *n* = 6	1.15 ± 0.21 *n* = 6	0.98 ± 0.11 *n* = 7	1.18 ± 0.11 *n* = 7	0.99 ± 0.07 *n* = 5	1.18 ± 0.18 *n* = 5

The levels of indicated proteins were analyzed with Western Blot method. Data were calculated as percentages of controls and are expressed as means ± SEM

*RS* restraint stress, *BET* betaxolol, *SAL* saline control

## Discussion

Here, we have presented the analyses of the influence of repeated RS on the expression of genes coding for Gα12/13 and Rho-GTPases in the PFC, AMY and HIP of mice, and the stress-induced effects on the expression of intracellular proteins related to the Gα12/13 signaling pathway in the rats HIP. In the latter case, we showed that repeated RS caused an increase in the RhoA level, that was normalized by betaxolol applied immediately after the stress procedure.

Previous studies have proven that Gαs, Gαi, Gαo, or Gαq/11 are engaged in the response to chronic stress or corticosterone administration [23–25]. In the current study, we found that repeated RS applied for 2 h daily decreased Gα12 mRNA expression both in the PFC and AMY. Gα13 expression, in turn, was reduced exclusively in the AMY, and none of the genes exhibited differential expression in the HIP after repeated restraint stress in mice. The discrepancy between the effects of stress on Gα12 and Gα13 mRNA expression is somewhat unexpected, as previous experiments using dominant active forms of Gα12 and Gα13 have demonstrated that these proteins signal through similar downstream effectors and, thus, likely have overlapping functions [26]. Nevertheless, studies have documented functional differences between these proteins by showing that Gα13 knock-out mice die in the uterus during embryonic development, while Gα12 knock-out mice exhibit almost no abnormalities [27]. What is more, a previous study indicating the differential distribution of Gα12 and Gα13 within neuronal cells (i.e. Gα12 localized in somata and neurites, whereas Gα13 mainly present in somata) [9], may suggest that Gα12 rather than Gα13 is engaged in synaptic remodeling. Proteins RhoA and Rac1 are representatives of the Rho-like and Rac-like families, two subfamilies of the Rho-family proteins. The limited studies concerning the role of Rho-family proteins in psychiatric disorders suggested that Rho-like and Rac-like subfamilies are involved in the pathomechanisms of schizophrenia, addiction and depression [28]. In our study, chronic RS in mice increased Rac1 mRNA expression in the PFC and decreased RhoA expression in the AMY.

All Rho-family proteins are downstream effectors of receptors coupled to Gα12/13 [29] and the expression of both heterotrimeric G proteins and small GTPases in response to chronic RS was simultaneously decreased in the AMY. However, in the PFC, the expression of Rac1 was increased, while Gα12 decreased. A possible explanation why Gα12 and Rac1 mRNA expression levels changed in opposite directions after restraint stress in the PFC may be based on likely interactions between Gα12/13 and Gαq/11 protein families [30, 30]. Rac1, in some instances, may be activated as a second messenger downstream of other G proteins, like Gq. Simultaneously, RhoA is generally the direct target of Gα12/13 [30], what explains why in the AMY decreases in the expression of RhoA and Gα12/13 were noted. No changes in mRNA expression were observed in the mice HIP. In contrast to our results, a previous in vitro study determined that treating amygdaloid and hippocampal cell lines with corticosterone results in increased RhoA mRNA levels but does not affect Rac1 expression [31]. However, this earlier study involved modeling stress by incubating cells with corticosterone for 24 h, which likely mimicked acute stress rather than chronic one. Nevertheless, in vitro or even in vivo corticosterone treatment probably does not fully reflect the myriad of different substances that can affect brain structure–function during stress treatment [32]. On the other hand, a previous study found decreased Rac1 mRNA expression in the nucleus accumbens of depressed patients and chronically stressed mice [28]. The lack of differential mRNA expression in the HIP we revealed in our study has been an intriguing issue. This structure has been recognized as a gateway to remodel brain structure and function by exposure to stress hormones [33].

Some data show that periods of stress shorter than 21 days are not sufficient to cause dendritic spines retraction in the HIP [34]. Moreover, Alfonso et al. [25] showed that the HIP stress caused altered expression of genes coding for molecules regulating neurite outgrowth, like NGF, BDNF, Gαq, after exposure to stress procedure more severe than 2 h daily. Indeed, when we applied chronic but not acute RS in rats for 3–4 h daily, we observed an increase of RhoA protein expression in the HIP. Rho-GTPases RhoA, RhoB, RhoC are ubiquitously expressed in mammals and possess 87% amino acid sequence identity; however, they have specific and pleiotropic intracellular functions [35]. In our study, chronic RS in rats upregulated only RhoA level. Our observation stays in agreement with the data showing high activation of RhoA in mice hippocampal slices exposed to corticotropin-releasing hormone (CRH) [36]. Similarly, it is significant that augmented RhoA activity was implicated in the mechanism of stress-evoked spine loss in dendrites of CA1 pyramidal neurons and was accompanied by LTP disturbance. Moreover, the authors revealed that other members of the Rho family of small GTPases are less affected by exposure to stress, what can explain the lack of RS-evoked changes we obtained in the expression of RhoB, RhoC and Rac proteins. In the HIP, the signaling proteins upstream to RhoA: Gα12 and cooperating with Gαq and Gα11, were not affected by stress in our study, suggesting that activation of Gα12/13-coupled receptors is not involved in the stress response in the rat hippocampus cells.

In our study, repeated RS resulted in the increase of RhoA protein level in the HIP of rats, while no change was observed in mRNA expression of the gene encoding for this protein in mice. The exact reason for the discrepancy between the effect of RS on RhoA mRNA and the RhoA protein level in the HIP of these two species is not clear. However, there a few arguments that seem plausible. First of all, it is known that the mRNA rate or degree of expression of a given gene does not necessarily reflect its protein expression level. Also, it is estimated that the protein expression level of only about one-third of proteins correlates with the level of their mRNA expression (when measured at the same time point). Both processes, the gene transcription and translation to protein, are subject to multi-level and complex regulations. In certain circumstances, the mRNA degradation may be enhanced to maintain mRNA homeostatic turnover. Thus, 24 h after the last stress session, when the brain tissue was isolated, the mRNA changes might be poorly detectable.

We are aware that our experimental approach has some limitations regarding the use of different species, i.e. mice and rats. Though both species are commonly used laboratory animals, their genetic diversity should be borne in mind. Furthermore, the regulation of gene expression depends indirectly on the activity of neural circuits. A certain difference between mice and rats may exist in the organization of brain circuits’ connectivity. For instance, Scheel et al. [37] described the interspecies differences regarding the integration of the nucleus reuniens (a part of the midline thalamus) in circuits of fear, aversion, and defense. The brain stress systems in rats and mice operate on the same bases, and across species there is some universal consistency with respect to brain regions expressing high levels of mineralocorticoid (MR) and glucocorticoid (GR) receptors in adulthood [38, 39]. However, these two species exhibit differences in the ontogenetic profiles of average MR and GR expression levels that may result in differential susceptibility to stress in adulthood [39].

We have shown for the first time that pharmacotherapy with betaxolol, the β1AR blocker, normalized the stress-evoked increase of RhoA level in the rat HIP. The β1AR are anatomically and functionally predisposed to respond to high noradrenaline concentration released during stress in the HIP. Therefore, these receptors could be implicated in stress-related plasticity. Immunohistochemistry revealed dense expression of β1AR in the HIP [40]. Electrophysiological studies showed their modulatory role in LTP occurrence in the HIP [41]. Betaxolol also alleviated stress symptoms in preclinical and clinical studies [21, 41]. However, the intracellular mechanism of plasticity in the hippocampus with β1AR involvement remains unknown. One possibility is a direct coupling of β1AR to G12/13, what has been recently suggested as a novel signaling pathway [42].

In conclusion, both protein families, Gα12/13 and Rho, were previously shown to be associated with brain neuroplasticity during nonpathological conditions [17, 30]. Our results suggest that these proteins are engaged in the mechanism of stress-related pathological changes in dendritic spines, as observed in other studies [5, 36, 43]. We have proven that betaxolol normalized the chronic stress-induced upregulation of RhoA in the HIP, indicating therapeutic usefulness of β1AR blockade in reversing stress-related disturbance of synaptic function and plasticity.

## Supplementary Information

Below is the link to the electronic supplementary material.

Supplementary file1 (DOCX 15 KB)

## Abbreviations

AMY: Amygdala
β1AR: β(1) Adrenergic receptors
BET: Betaxolol
GTPase: Guanosine-5ʹ triphosphatase
HIP: Hippocampus
5-HT: Serotonin
PFC: Prefrontal cortex
*po*: Oral administration (lat. per os)
Rho protein: The Ras homologous (Rho) protein family
RS: Restraint stress
SEM: Standard error of mean
