# Supplementary material for: Chronic restraint stress induces changes in the cerebral Galpha 12/13 and Rho-GTPase signaling network
Source: Pharmacol Rep. 2021 Jun 11;73(4):1179–87. doi: 10.1007/s43440-021-00294-4 (PMC8413188; doi:10.1007/s43440-021-00294-4)
Supplement: Supplementary file 1 — Supplementary file1 (DOCX 15 KB) [file 43440_2021_294_MOESM1_ESM.docx]

Chronic restraint stress induces changes in the cerebral Galpha 12/13 and Rho-GTPase signaling network

Katarzyna Rafa-Zabłocka^1#^, Agnieszka Zelek-Molik^1#^, Beata Tepper^2^, Piotr Chmielarz^1^, Grzegorz Kreiner^1^, Michał Wilczkowski^1^ and Irena Nalepa^1*^

**^1^** Department of Brain Biochemistry, Maj Institute of Pharmacology, Polish Academy of Sciences, Smętna 12, 31-343 Kraków, Poland; **^2^** Laboratory of Calcium Binding Proteins, Nencki Institute of Experimental Biology, Polish Academy of Sciences, Pasteura 3, 02-093 Warsaw, Poland

Supplementary Table 1. Summary of the two-way ANOVA results showing RS effects, BET effects and RS×BET interaction effects on the protein expression of Gα(12), Gα(11), Gα(q), Rho B, Rho C, Rac 1/2/3 in the rats’ hippocampus.

|  | | Acute Restraint Stress | | | Chronic Restraint Stress | | |
| --- | --- | --- | --- | --- | --- | --- | --- |
| Name of protein | Source of variation | Degr. of Freedom | F | p | Degr. of Freedom | F | p |
| Gα(12) | RS | 1,20 | 2.66 | 0.12 | 1,19 | 0.57 | 0.46 |
|  | BET | 1,20 | 0.01 | 0.91 | 1,19 | 0.23 | 0.63 |
|  | RS×BET | 1,20 | 0.97 | 0.34 | 1,19 | 0.37 | 0.55 |
| Gα(11) | RS | 1,17 | 0.05 | 0.83 | 1,19 | 0.00 | 0.95 |
|  | BET | 1,17 | 1.07 | 0.31 | 1,19 | 0.16 | 0.69 |
|  | RS×BET | 1,17 | 0.52 | 0.48 | 1,19 | 0.10 | 0.75 |
| Gα(q) | RS | 1,20 | 0.97 | 0.34 | 1,20 | 2.27 | 0.15 |
|  | BET | 1,20 | 0.59 | 0.45 | 1,20 | 0.13 | 0.71 |
|  | RS×BET | 1,20 | 2.93 | 0.10 | 1,20 | 0.54 | 0.47 |
| Rho B | RS | 1,20 | 1.00 | 0.33 | 1,20 | 0.05 | 0.82 |
|  | BET | 1,20 | 0.46 | 0.51 | 1,20 | 1.31 | 0.27 |
|  | RS×BET | 1,20 | 0.07 | 0.79 | 1,20 | 0.67 | 0.42 |
| Rho C | RS | 1,20 | 0.28 | 0.60 | 1,18 | 0.96 | 0.34 |
|  | BET | 1,20 | 0.02 | 0.88 | 1,18 | 1.80 | 0.20 |
|  | RS×BET | 1,20 | 1.41 | 0.25 | 1,18 | 0.17 | 0.68 |
| Rac1/2/3 | RS | 1,20 | 0.30 | 0.59 | 1,20 | 2.54 | 0.13 |
|  | BET | 1,20 | 0.06 | 0.80 | 1,20 | 0.00 | 0.98 |
|  | RS×BET | 1,20 | 0.08 | 0.78 | 1,20 | 0,00 | 0.96 |

RS – restraint stress; BET – betaxolol; SAL- saline
